# Reduction malarplasty using a simulated surgical guide for asymmetric/prominent zygoma

**DOI:** 10.1186/s13005-022-00314-5

**Published:** 2022-03-29

**Authors:** Sang-Hoon Kang, Hye-Jin Tak, Hak-Jin Kim, Sang-Hwy Lee

**Affiliations:** 1 Department of Oral and Maxillofacial Surgery, College of Dentistry, Yonsei University, Seoul, Republic of Korea; 2 Department of Oral and Maxillofacial Surgery, National Health Insurance Service Ilsan Hospital, Goyang, Republic of Korea; 3 Oral Science Research Center, College of Dentistry, Yonsei University, Seoul, Republic of Korea; 4 Private Dental Clinic, Yongin, South Korea; 5 Department of Oral and Maxillofacial Surgery and Oral Science Research Center, College of Dentistry, Yonsei University, 50-1 Yonsei-ro, Seodaemun-gu, 03722 Seoul, Republic of Korea

**Keywords:** Zygoma, Reduction, Surgical guide, Asymmetry, Prominency, Computer-assisted surgery, CAD/CAM

## Abstract

**Background:**

The present study introduces a reduction malarplasty using a three-dimensional (3D)-printed surgical guide and evaluates the guide’s technical applicability.

**Methods:**

Twenty malarplasties were performed for 12 subjects with zygomatic asymmetry/prominency using the current method. 3D reconstruction of the craniomaxillofacial region and fine dental occlusion was made with image data from computed tomograpy and dental scanning. A computer-assisted surgical simulation was performed for reduction malarplasty and a surgical guide was designed for later 3D printing. The manufactured surgical guide was introduced to the operation field to guide the surgery; its surgical accuracy was confirmed by comparing five corresponding points from preoperative simulation and postoperative data.

**Results:**

We successfully performed the reduction malarplasty with the surgical guide. The accuracy level of surgery fell to 0.93 mm of total median difference for the corresponding zygoma points of preoperative simulations and postoperative zygoma. The anterior and upper points showed less error level (0.59 and 0.73 mm difference, respectively) than did other points.

**Conclusions:**

We developed a computer-assisted surgical technique using a surgical guide for asymmetrical/prominent zygoma which proved to be simple, practical, and accurate; it is expected to help surgeons perform reduction malarplasty with ease and accuracy.

## Background

Recent advances in computer-assisted surgery allow three-dimensional (3D) orthognathic analysis, surgical simulation, and 3D printing of surgical guides using computed tomography (CT) images during the treatment of deformity or facial asymmetry [1]. For malarplasty, preoperative planning based on appropriate diagnosis and surgical simulation is crucial to obtain esthetically and functionally satisfactory results [1, 2]. Computer-assisted surgical simulation and the production of surgical guides by computer-assisted design (CAD)/ computer-assisted manufacturing (CAM)-engineered 3D printing clearly facilitate accurate osteotomy and/or reduction of zygomatic bone.

The malar, or zygomatic, bone has a body and three extensions of bony process, articulating with the frontal, sphenoid, temporal, and maxillary bones. It is located at the intersection of important functional units, including vision, upper respiratory tract, and masticatory complex [3]. It has the muscular attachment of masseter muscle, and its zygomatic arch and temporal bone form a perforated space for temporal muscle. It projects medio-laterally at the upper lateral face to form the prominency of cheekbone, and is frequently asymmetrical in shape and size [4, 5].

It is therefore essential to achieve the proper amount of surgical reduction and balanced symmetrical prominence of postoperative zygoma for the treatment of asymmetric/prominent zygoma. Traditional (and currently popular) reduction malarplasty demands a high level of expertise and precision to accomplish predictable and symmetrical reduction of zygoma [6]. In addition, the currently available reduction method requires a relatively greater amount of bony reduction to achieve a moderate reduction in facial-zygomatic appearance, since the bony reduction at the broad zygomatico-maxillary junction is far from the rotation center of the proximal zygomaticotemporal junction. It also fails to provide sufficient physical and visual access to the zygomaticotemporal junction, where the loaded stresses are concentrated and fixation is recommended [7].

CAD/CAM-based surgical guides for malarplasty allow the shape, height, and curve of the osteotomy line to be made accurately and adjusted easily with consideration for changes in facial soft tissue. The osteotomy line can be planned and applied more delicately, preventing damage to the surrounding anatomical structures by referring to CT images and the location of the zygomatic nerves. It can benefit both experienced and junior surgeons regardless of the type of facial bony plastic surgery.

This paper introduces our reduction malarplasty for asymmetric/prominent zygoma and reviews its accuracy based on surgical simulation and a CAD/CAM guide with or without bimaxillary orthognathic surgery.

## Methods

**Fig. 1:**
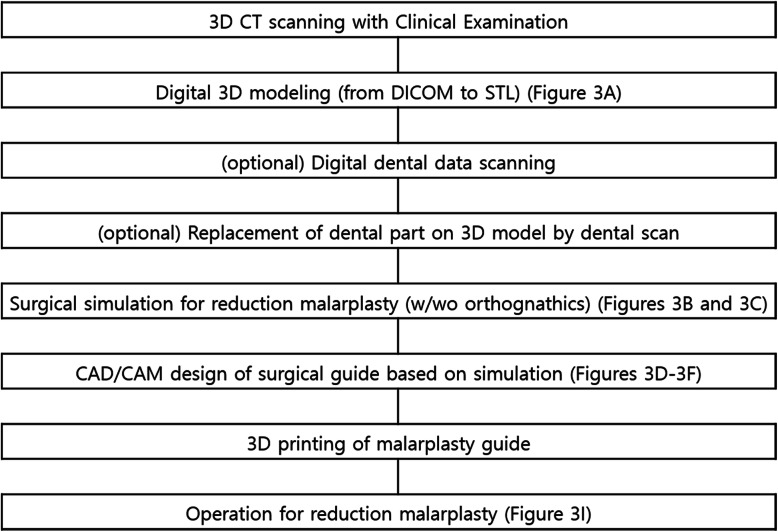
A flow chart for preoperative preparation, including the surgical guide design. Please see related figures described at the end of each step for further references

**Fig. 2:**
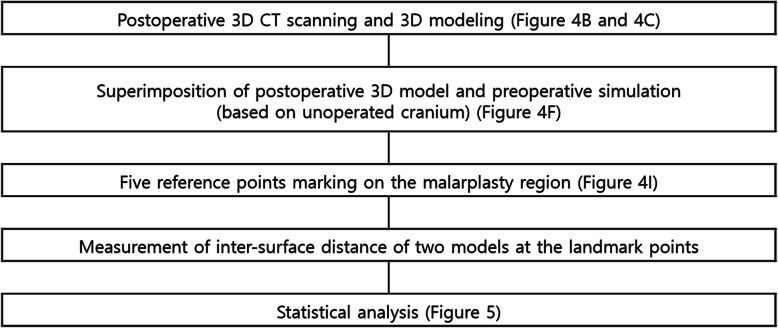
A flow chart for post-malarplasty accuracy evaluation, performed in this study. Please see related figures described at the end of each step for further references

All subjects had reduction malarplasty independently or simultaneously with orthognathic surgery. As described below, the subjects underwent 3D surgical diagnosis for facial deformity/asymmetry and surgical simulation, the surgical guide then being designed using CAD/CAM and 3D printing. The process of the preoperative preparation and postoperative validation is introduced in flow charts (Figs. 1 and 2). This study was approved by the Institutional Review Board.

### Surgical simulation and production of a 3D-printed CAD/CAM surgical guide for reduction malarplasty in facial asymmetry

The facial CT data were obtained with less than 1 mm of slice thickness and 512 × 512 of image resolution, then reconstituted into a 3D image set by importing Digital Imaging and Communications in Medicine (DICOM) files into Mimics (version 18.0, Materialise, Leuven, Belgium). A 3D skeletal model was produced to include zygoma and maxillofacial structure.

**Fig. 3:**
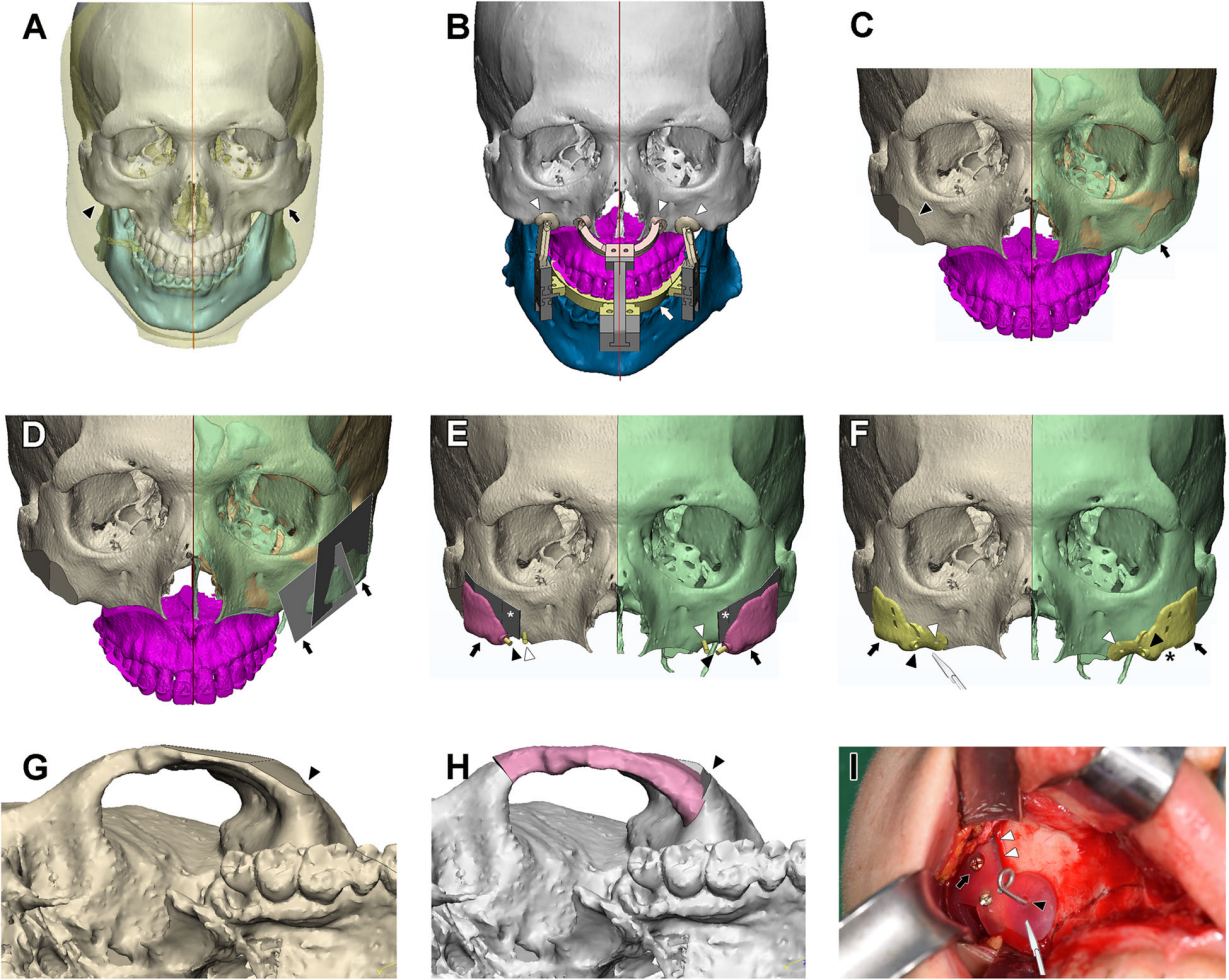
**A **Pre-operative image of three-dimensional craniofacial skeletal and skin models with midsagittal plane. The more prominent left zygoma (indicated by arrow) in relation to the right zygoma (indicated by arrowhead) reveals the asymmetric configuration. Red line for midsagittal plane; white for craniomaxilla; light blue for mandible; transparent yellow for facial skin. **B** Three-dimensional (3D) surgical simulation made on the maxilla (purple pink) with preoperative mandible (dark blue). The computer-assisted surgical guides, including interocclusal wafer (yellow; indicated by arrow) and Y- and zygoma-guide (dark ivory; indicated by arrowheads) were designed by 3D cephalometry and CAD/CAM technique for bimaxillary orthognathic surgery. Red line for midsagittal plane; purple pink for postoperative maxillary segment; dark blue for preoperative mandible; gray and dark ivory for Y-guide. **C** The asymmetric and more prominent zygoma on the left side (transparent light green; indicated by arrow) was superimposed with the mirrored right zygoma to left (yellow) after initial cutting on the right zygoma (dark ivory; indicated by arrowhead) based on the treatment plan. Red line for midsagittal plane and used for mirroring; purple pink for postoperative maxillary segment. **D** The double osteotomy lines (gray planes; indicated by arrows) were placed on the left zygoma (transparent light green) for the simulated reduction malarplasty to produce the symmetrical and balanced zygoma based on the treatment plan and the superimposed mirror image of right zygoma (yellow). Red line for midsagittal plane, used for mirroring; purple pink for postoperative maxillary segment; mirrored right zygoma to left side (yellow); right zygoma with zygomatic cuts (dark ivory). **E** The draft of the surgical guide (pink; indicated by arrows) was designed to have double osteotomy lines (gray bent planes; indicated by asterisks) and fixation screws (yellow cylinder 1; indicated by black arrowhead) and a reference hole guide (yellow cylinder 2; indicated by white arrowhead). Right zygoma (dark ivory); left zygoma (light green). **F**The final desgin of the CAD/CAM-based surgical guide (indicated by arrows) for reduction malarplasty with cutting slots for osteotomies (indicated by saw blade), fixation holes for stabilizing the device with screws (indicated by white arrowhead), a reference hole guide (indicated by arrowhead) for the placement of the guide, and the window opening (asterisk) to check how well the device fits to the zygomatic bone surface. **G** and **H** The morphological comparison of zygoma (indicated by arrowheads) after the simulated reduction malarplasty by our new development (in Fig. 3G) and the traditional technique (in Fig. 3H). The osteotomized zygoma of traditional technique in Fig. 3H was moved to the extent that it could match the zygomatic surface made by our reduction malarplasty in Fig. 3G. **I** An intraoperative view of a CAD/CAM surgical guide placed in situ with osteotomy slot (indicated by two white arrowheads and guided by saw blade), fixation screws (arrow) and reference guide pin (black arrowhead) before performing the reduction malarplasty

The maxillary and mandibular dental models were scanned using the optical 3D scanner Rexcan DS2 (Solutionix, Seoul, Korea), their stereolithography (STL) model data being produced for a digital dental model. Using the 3-matic program (version 10.0, Materialise, Leuven, Belgium), the maxillary and mandibular teeth on the 3D skeletal model were replaced by the digital dental model using sequential point- and volume (or surface)-based registration (Fig. 3 A) [8].

The orthognathic surgical simulation was performed to set an appropriate maxillary/mandibular position and facial shape based on clinical evaluation as well as on 3D cephalometry [9], and to construct final occlusion, which was manually set and digitally scanned on the plaster models, and the midsagittal plane, which was constructed with three landmarks and confirmed clinically [10, 11]. The mandible was first set using the predicted postoperative final occlusion and the maxillomandibular complex was positioned for proper skeletal symmetry and balance during the simulation [9]. A horseshoe-shaped orthognathic surgical guide or wafer was designed based on the simulated preoperative mandibular and postoperative maxillary occlusion. An orthognathic maxillary cutting guide was also designed to enable the maxillary cut and interference removal for maxillary repositioning at the maxillary anterior wall near the zygomatic cut (Patent cooperation treaty (PCT) KR2014/010282; Korean patent 10-1478009) [12]. The reference hole for repositioning the maxilla also served to place the cutting guide for reduction malarplasty. During the orthognathic surgery, a 3D-printed surgical wafer was placed after the Le Fort I osteotomy of the maxilla, the 3D-printed cranium-based surgical guides then being connected for more precise movement of the down-fractured maxillary segment to the predicted position (Fig. 3B; PCT KR2014/010284; Korean patent 10-1501447) [13].

The malar shape and its symmetry were evaluated to perform zygomatic simulation surgery with and without orthognathic simulation. The less prominent side of the zygoma was mirrored to the other side along the midsagittal plane and their shapes were compared (Fig. 3 C). The midsagittal plane was manually constructed based on three landmarks, including the orbit and center of the foramen magnum, and clinically adjusted if necessary [11]. The more prominent area of the zygomatic body was positioned for surgical cut, and a one- or two-cutting plane was designed using 3-matic software (Fig. 3D). Care was taken not to expose the maxillary sinus, not to break the continuity of the zygomatic process, and not to damage the zygomaticofacial nerve.

The reduction malarplasty was simulated in a 3D model based on the previously designed cutting plane and the surgical guide outline was designed with cylinders positioned for the reference and stabilizing screws or pins (Fig. 3E). The osteotomy could be simulated using the cutting plane, thus controlling the location and depth of the osteotomy and therefore the amount of bone removal. The surgical guide was designed with careful consideration of device stability and intraoperative visibility. It covered the zygomatic and upper maxillary area and had a cutting slot for accurate cutting location and direction (Fig. 3 F). It also had one or two screw holes for the immobilization of the guide on the zygoma, particularly during the sawing procedure. It had an optional design of shelf-shaped foot-extension, which was to be hooked on the superior border of the zygomatic arch and/or medial side of the zygomaticofrontal process for accurate positioning without a reference point. It can have an additional one foot-extension with a screw hole located at the same reference point produced by the previously described maxillary cutting guide to assist in accurate positioning of this guide in simultaneous orthognathic surgery. The morphological comparison of zygoma after the simulated reduction malarplasty was performed by our new development (Fig. 3G) and the traditional technique (Fig. 3 H).

The zygoma guide without a common reference hole could be positioned based on the superficial contour of the maxillary and zygomatic bones, assisted by the previously described hooking stability of the device’s small extension onto the zygomatic arch or zygomaticofrontal process. It also had a window opening at the lower border to confirm proper positioning by observing intimate contact of the guide with the zygomatic bone. The designed surgical guide was exported in STL data format for 3D printing with a biocompatible material (ProJet 3500 HDMax 3D Printer, 3D Systems, Inc, Rock Hill, SC). The device design was registered to Korean patent (10-1514237).

### Reduction malarplasty using simulated surgical guide

The surgical guide for the zygomatic reduction was placed in the zygomatic area after exposing the surgical field by extended intraoral vestibular incision and dissection, stability and conformity to the preplanned location having been confirmed. One or two screws or pins 6–8 mm in length were introduced to fix the guide onto the zygomatic bone surface (Fig. 3I). The osteotomy line was pre-drawn by surgical drill to allow for positioning of a pre-planned osteotomy line while the surgical guide was monitored for inadvertent movement. Controlled reciprocal sawing was performed using the tailored cutting slot of the surgical guide, cut depth being controlled by the length of the saw blade. The surgical guide was removed after the initial sawing, additional reciprocal sawing being performed in the osteotomy line. The zygomatic bone segment was finally cut and retrieved by applying the osteotome, the sharp bony edges around the osteotomy line then being trimmed with burs.

### Setting measurement points and measuring errors

**Fig. 4:**
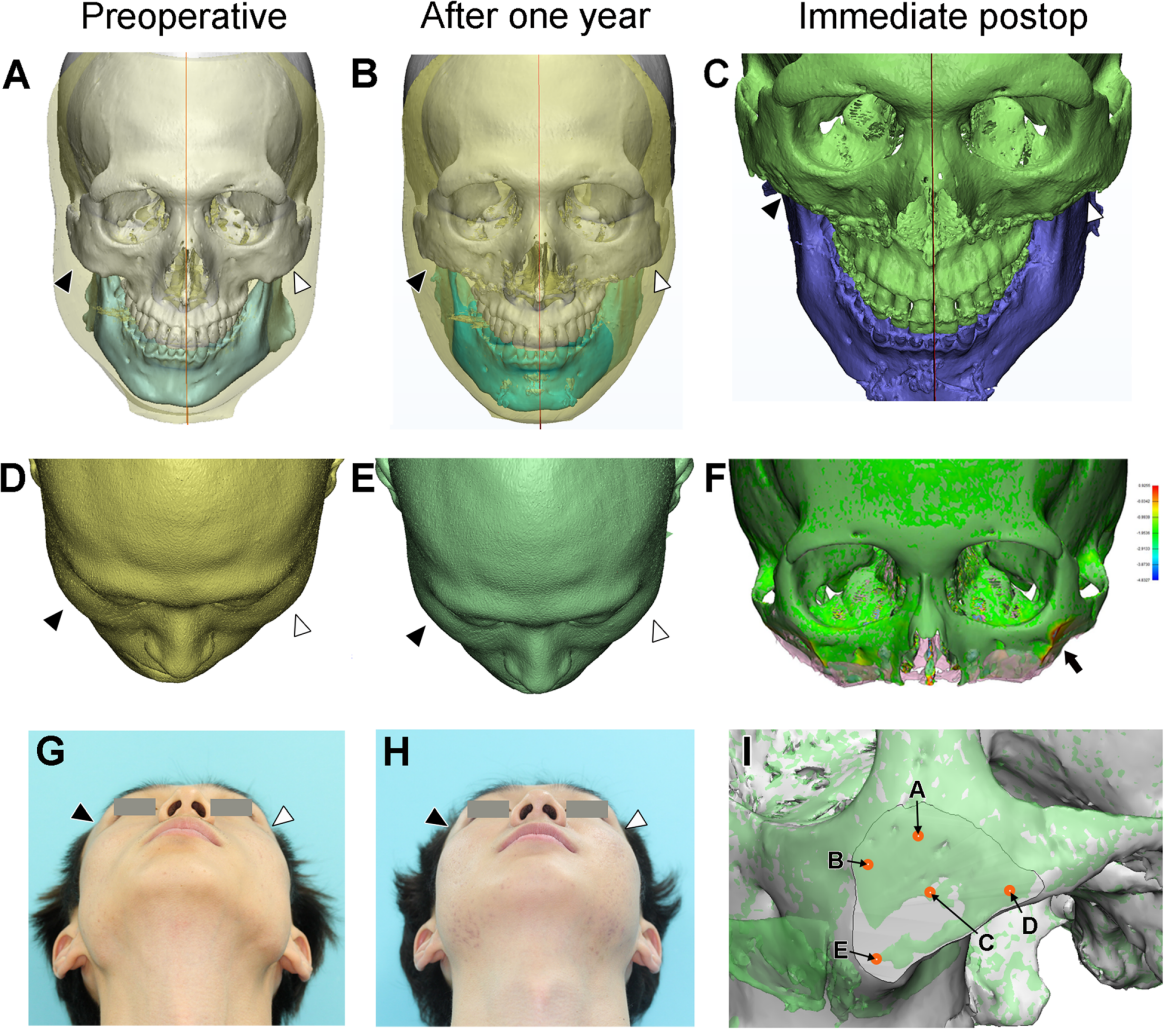
**A-C** The preoperative, immediate and one-year postoperative three-dimensional images of maxillofacial region before and after reduction malarplasty with orthognathic surgery, showing the prominent zygomas preoperatively (black and white arrowheads in **A**), immediate postoperative shape (arrowheads in **C**), and their postoperative appearance after one year (arrowheads in **B**). **D **and** E** The morphological comparison of the preoperative (**D**) and the one-year postoperative (**E**) craniofacial region, being focused on the contour of the zygoma (indicated by the white and black arrowheads), from the same subject of the simulation and surgery in Fig 3 and 4. **F** Color-coded discrepancy map comparing the simulated and immediate postoperative 3D model for reduction malarplasty, showing the limited level of their discrepancy (as indicated by arrow). The superimposition accuracy of simulation and postsurgical models, measured as the inter-surface average distance of two cranial models, was 0.13 ± 0.09. **G **and** H** Comparison of the preoperative (Fig 4D) and the one-year postoperative (Fig. 4E) facial appearances, being focused on the contour of the zygoma (indicated by the white and black arrowheads), of the same subject for simulations and surgery in Figs. 3and 4. **I** The superimposition of preoperative simulation model (in gray) and the postoperative model (in transparent light green) to show the left malarplasty region. Five reference points (marked as white points surrounded by orange circles and indicated by arrows) were marked on the surface of postoperative light green model: center (**C**), upper (frontal;**A**), lower (maxillary; **E**), anterior (orbital; **B**), and posterior (zygoma arch;**D**). Average distances between two corresponding reference points of the simulation and postoperative 3D models ranged between 0.59 and 1.42 mm, median distances for all five points reaching 0.93 ± 0.64 mm

Measurements were made at several regional points of the zygoma to evaluate surgical accuracy and morphological differences between the preoperative simulation and postoperative 3D models (Fig. 4 A-E). The simulated and operated 3D craniofacial models of a subject were first superimposed based on the surface registration of the unchanged cranial part (Fig. 4 F).

For comparison of the preoperative (Fig. 4G) and the one-year postoperative (Fig. 4 H) facial appearances, being focused on the contour of the zygoma, of the same subject for simulations and surgery, five reference points were marked on the zygomatic surface of a 3D model: center (C), upper (frontal; A), lower (maxillary; E), anterior (orbital; B), and posterior (zygoma arch; D), based on preoperative planning and immediate postoperative evaluation (Fig. 4I). The shortest 3D distance in absolute value between the two models at a reference point was calculated as the length of a normal line drawn from the reference point of a model to the surface of another model by software function.

One of the authors (KSH) did all the pointing on and measurements of the surgical simulation and postoperative models in order to avoid inter-individual errors. 3D distance between corresponding points from two models was considered surgical error in our reduction malarplasty. We analyzed such errors and verified their significance by Kruskal-Wallis test with a significance level of 0.05, Dunn’s multiple comparisons test, and Bland-Altman analysis using IBM SPSS Statistics 23 (IBM Corp., Armonk, NY, USA).

In order to measure possible errors arising from the measurement process as method error, one author repeatedly (10 times) set a reference point at the zygoma center. In addition, another possible methods error, which could develop during the superimposition process of the cranial structures, was evaluated: two identical cranium models of 5 different subjects were superimposed independently using the same method in this study, and the inter-surface distances were measured and statistically analyzed.

## Results

CAD/CAM surgical guides for reduction malarplasty were applied to 12 subjects (20 cases) with asymmetric/prominent zygoma. The mean age (± standard deviation (SD)) was 23.0 ± 5.84 years old and the female-to-male ratio was 10:2. Bilateral reduction malarplasty was done for eight subjects, unilateral for four subjects. All twenty cases of reduction malarplasty were performed using the CAD/CAM surgical guide based on computer-assisted surgical simulation.

This zygomatic reduction surgery was performed independently or simultaneously with maxillary orthognathic surgery. We were able to easily place an accurate zygomatic osteotomy line and perform the reduction by extending the maxillary vestibular incision. The extent of bone reduction for a symmetrical and balanced zygoma could be obtained as planned without any complication (Fig. 4 A-C). Facial appearances were also improved in the zygomatic region (Fig. 4D, E, G, and H).

**Fig. 5:**
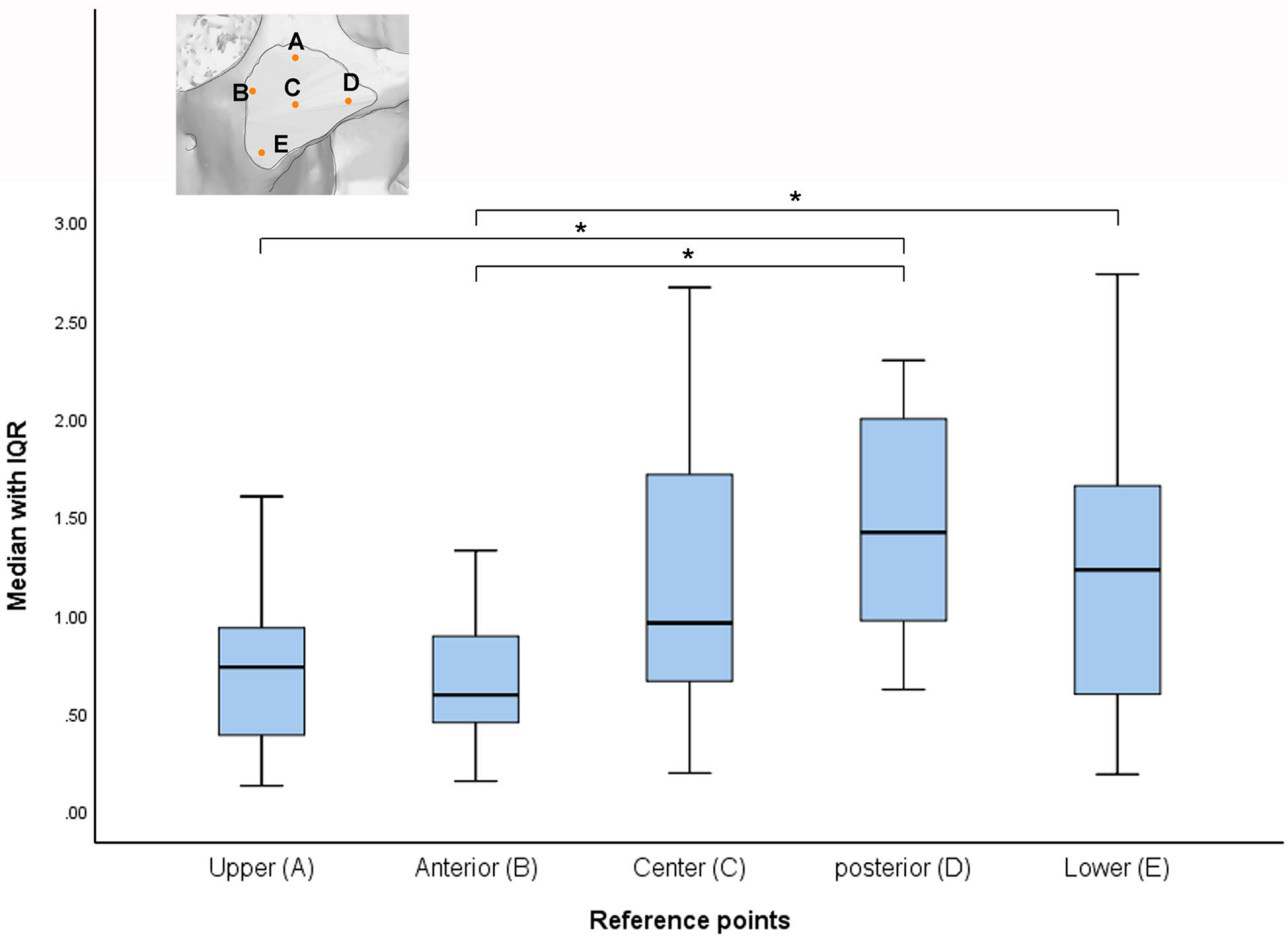
Three-dimensional distances between the zygoma reference points produced on presurgical simulation and postsurgical models (*N* = 20 for each point). The measurements at each point were displayed in boxplots with median, maximum, and minimum, with their upper and lower quartiles, to indicate the higher accuracy level at the point (**A** and **B)**. The statistical analysis and comparison was performed by Kruskal-Wallis test, and Dunn’s comparison test (* p < 0.05), as shown in the figure. The measured reference points were designated as follows: Center (**C**) at the zygoma center; Upper (**A**) at the zygoma upper area for frontal direction; Lower (**E**) at the zygoma lower area for maxillary direction; Anterior (**B**) at the zygoma anterior area for orbital direction; Posterior (**D**) at the zygoma posterior area for zygoma arch direction

Average distances between two corresponding reference points of the simulation and postoperative 3D models ranged between 0.59 and 1.42 mm, median distances for all five points reaching 0.93 ± 0.64 mm (Fig. 5). The differences varied significantly depending on location (*p* < 0.0001 by Kruskal-Wallis test), but not between the simulation and postoperative models (details not shown; Bland-Altman analysis; 0.154 < *p* < 0.756 and − 0.377 < bias < 0.004). The difference at the anterior point (orbital area) was 0.59 ± 0.33 mm in median, significantly smaller than that at the posterior point (zygomatic arch area; 1.42 ± 0.58 mm) (*p* = 0.0005) or lower point (maxillary area; 1.23 ± 0.72 mm) (*p* = 0.0411). In addition, the difference at the posterior point (zygomatic arch) was also significantly greater than at the upper point (frontal area; 0.73 ± 0.39 mm) (*p* = 0.0014).

The superimposition accuracy of simulation and postsurgical models, measured as the inter-surface average distance of two cranial models, was 0.13 ± 0.09. Intra-examiner error was measured by calculating the differences in the position of marked points of the zygoma center, which showed 0.35 ± 0.23 mm.

## Discussion

The malar bone or zygoma possesses functional and structural roles; being located at the center of several cranial functional units, it has functional roles in mastication, resistance to bending/tension force, and orbital frontation and convergence [3]. It is also one of the main skeletal components affecting general facial appearance. A prominent zygoma with curved surface gives a relatively strong or muscular impression, while a smoothly rounded zygoma appears softer. In order to reduce the prominency of zygoma, several techniques, mainly via intraoral and/or facial approach, have been advocated, including bone shaving, I- or L-shaped osteotomy, and wedge-shaped osteotomy on the zygomatic body and process [14].

Postoperative complications related to these reduction malarplasties are widely reported and include infection, sensory change, cheek drooping or ptosis, and mal- or non-union of fragments [6]. Our reduction malarplasty was performed with easy and accurate placement of the osteotomy line without noticeable complications. Though those popular malarplasty techniques focus on the reduction of bony zygoma at the highest prominency (or summit of zygoma), they fail to guarantee it [15]. The techniques cannot properly predict the amount of zygomatic prominency reduction, nor ensure intersegmental bony union or postoperative balanced shape.

The results of our evaluation of the accuracy level of reduction malarplasty with cutting guide seem good enough: the cutting surfaces of the planned and postsurgical models matched quite well with only a small difference (0.93 ± 0.64 mm in median). This means we can perform this reduction malarplasty with predictable results.

However, this accuracy level was different depending on the location: the farther from the anterior starting point, the greater the level of inaccuracy. This may be due to the application of the cutting guide: the accuracy at the anterior and upper point was far higher than that at the posterior and lower point. This is to be expected in that the execution of osteotomy along the slot of the cutting guide gains accuracy as it gets near the starting point and closer to the fixation screws and reference hole. As it gets farther from the reference hole and screws, we may expect greater deviation from the planned osteotomy line. The only exception to this tendency is the lower point, which is located in the anterior region near the screws or hole. This could be due to edge-grinding at the bone surface of the osteotomy line to smooth the bone surface. The greater difference may be due to the simulation not including edge-grinding.

We are therefore convinced that our malarplasty can achieve a predictable reduction of zygoma to yield a balanced postoperative zygoma without facial asymmetry. The cutting guide was placed by the bone-surface-matched contour as well as an additional reference hole which was produced by the cutting guide for maxillary surgery. The design of the guide, though simple, included almost all components necessary to perform the zygoma shaving.

The limited surgical field of vision to place the guide was avoided by sufficiently exposing the surgical site via extended vestibular incision and was fine after placement. Another potential problem relates to the exposure of the maxillary sinus or zygomatic arch fracture, which could be avoided in all cases by delicate simulation and operation. The superimposed 3D models of postoperative and simulation CT data showed a high level of compliance, confirming the accuracy of our method (Fig. 4 F). These points support the utility of this method for reduction malarplasty. Therefore, particularly in case of facial asymmetry with asymmetric/prominent zygoma, the introduction of computer-aided surgical simulation and the CAD/CAM surgical guide merit consideration.

The development of surgical guides for orthognathic or facial surgery has been recently reported [1, 12, 13, 16]. A cutting guide for orthognathic surgery has many advantages, such as the enhanced ability to adjust the shape of the osteotomy line and to predict the amount and region of prematurity, while minimizing operation time and possible damage to vital structures [17, 18]. Guides can be used for bone removal and/or osteotomy of the jaw, in conjunction with the positioning of jaws and measuring the jaw movement range. Moreover, the introduction of various computer-assisted surgical techniques, such as navigational or robotic surgery, hold promise for further advances in malarplasty [2, 19, 20].

This method can be applied to different surgical techniques for malarplasty with multiple bone cuttings [21]. However, without a CAD/CAM surgical guide, the simulation data alone cannot guarantee high accuracy of bone cutting or reduction, due to erroneous angulation or placement of the cutting instrument. Also, though the surgical guide can be produced manually from a rapid-prototyped model without CAD/CAM technology, it may be difficult to attain a symmetrical zygoma or to correct the cutting path or plan.

Surgical guides, however, have their shortcomings: they need to be designed and produced before operation using computer-assisted surgical techniques as well as 3D printers and medical materials; all these require the introduction of software, CAD/CAM technology and medicolegally-approved printing materials [22]. Attention also needs to be paid to preoperative surgical simulation, which must be done sensibly and accurately and then completely confirmed in the operation room. A surgical guide based on poor simulation may complicate the surgical procedure, yielding adverse results. CT scans with low resolution and/or greater slice thickness will produce an inaccurate zygoma model that will inevitably lead to inaccurate simulation and cutting guide fabrication, yielding poor surgical results of malarplasty.

Additional factors must be considered in designing the surgical guide, including its rigidity and durability. A thin guide cannot sustain the force exerted on it. If it is too thick, however, it may disturb the visual range of the surgical field. Moreover, the particles generated from the resin material of the guide during the cutting process may remain on the site, irritating the tissue or causing an allergic reaction. Though the guide is made of biocompatible material, it should not remain in the body.

## Conclusions

A CAD/CAM-based surgical guide for reduction malarplasty secured surgical accuracy and facilitated surgery in the zygomatic area for the treatment of asymmetric/prominent zygoma. It also ensured a good field of vision due to its small size and more accurate placement by virtue of dictating the superficial contour and sharing the reference points produced by the cutting guide. In addition, its simple design helped streamline the complicated design/production process. Development and testing of surgical guides will continue for facial bony plastic surgery.

## Data Availability

The datasets used and/or analyzed during the current study are available from the corresponding author upon reasonable request.
